# Planktonic and Benthic Bacterial Communities of the Largest Central European Shallow Lake, Lake Balaton and Its Main Inflow Zala River

**DOI:** 10.1007/s00284-020-02241-7

**Published:** 2020-10-17

**Authors:** Milán Farkas, Edit Kaszab, Júlia Radó, Judit Háhn, Gergő Tóth, Péter Harkai, Árpád Ferincz, Zsófia Lovász, András Táncsics, Lajos Vörös, Balázs Kriszt, Sándor Szoboszlay

**Affiliations:** 1 Department of Environmental Protection and Safety, Szent István University, Páter Károly utca 1, Gödöllő, 2100 Hungary; 2 Department of Aquaculture, Szent István University, Páter Károly utca 1, Gödöllő, 2100 Hungary; 3 Department Kis-Balaton, West-Transdanubian Water Directorate, Csík Ferenc sétány 4, Keszthely, 8360 Hungary; 4 Regional University Center of Excellence in Environmental Industry, Szent István University, Páter Károly utca 1, Gödöllő, 2100 Hungary; 5 Balaton Limnological Institute, Centre for Ecological Research, Klebelsberg Kuno utca 3, Tihany, 8237 Hungary

## Abstract

Lake Balaton is the largest European shallow lake, which underwent cultural eutrophication in the ‘70–80s. Therefore, strict pollution control measures were introduced and the water quality has become meso-eutrophic since the millennium. Due to the touristic significance and change in trophic levels of the lake, numerous ecological studies were carried out, but none of them was focused on both benthic and planktonic microbial communities at the same time. In our study, an attempt was made to reveal the spatial bacterial heterogeneity of the Lake Balaton and Zala River by 16S rDNA terminal restriction fragment length polymorphism fingerprinting and Illumina amplicon sequencing methods in the summer of 2017. According to the molecular biology results, mostly well-known freshwater microorganisms, adapted to nutrient-poor conditions were found in the pelagic water column. The LD12 subclade member *Fonsibacter ubiquis*, the cyanobacterial *Synechococcus* sp. and unknown Verrucomicrobia species were abundant in the less nutrient-dense basins, while the hgcI clade members showed various distribution. In the estuary and in the nutrient-dense western part of the lake, some eutrophic conditions preferring cyanobacteria (filamentous *Anabaena* and *Aphanizomenon* species) were also detectable. The benthic microbial community showed higher diversity, according to the observed appearance of microorganisms adapted to the deeper, less aerated layers (e.g. members of Desulfobacteraceae, Nitrosomonadaceae).

**Electronic supplementary material:**

The online version of this article (10.1007/s00284-020-02241-7) contains supplementary material, which is available to authorized users.

## Introduction

Lake Balaton, with a surface area of 594 km^2^ and an average depth of 3.2 m, is the largest central European shallow lake. The Balaton lakebed is 2–3 m deep at the north coast and gradually drops towards the south. The deepest points are usually located 1–1.5 km from the south shoreline where the shallow coastal zone (1–1.5 m) deepens relatively quickly down to 4 m depth. The upper sediment (10–30 cm) is generally very soft colloidal, whereas, due to the wave breaking effect, large grain sediment is observable at the shallow part of the southern coast. Both sediment types are well aerated and frequently suspended during strong wind events [1]. A total of 51 watercourses feed the Balaton, but only 20 have a permanent discharge. Among them, the largest Zala River, with 6 m^3^/s average discharge, drains the 45% of the catchment area and contributes to approximately half of the total phosphorus and nitrogen load of the lake. The sole outflow is a highly regulated channel at the eastern part, while the Zala River discharges to the westernmost part of the lake, which results in a trophic gradient from west to the east.

Due to the high external nutrient loads, the lake became hypertrophic in the ‘70s, and a west–east gradient of trophic state developed, and therefore strict water conservation regulations were introduced. Additionally, the Kis-Balaton Water Protection System was designed on the lower part of River Zala. The first stage of the system was introduced in 1985, while the second has been partly operating since 1992. Finally, for the more efficient removal of nutrients and flexible operation opportunities, modifications were accomplished in the second phase between 2011 and 2014. In the last decades, due to the beneficial changes in agriculture, wastewater treatment and the nutrient retaining effect of the water protection system, a reoligotrophication was observable and the values stayed between meso- and eutrophic.

Nowadays, climate change (i.e. warming and rising of evaporation) is a rising problem of concern for the life of the lake. Between the years 2000 and 2017, there were 7 years of negative water balance in the water supply of the Balaton. Therefore, the summer water level was artificially raised with 10 cm with an allowed fluctuation of 5 cm. The impact assessments have shown that regulation can be beneficial for tourism and agriculture. However, the test run has also highlighted the fact that reed swamps, which are ecologically important and ensure good water quality of Lake Balaton, require more water level fluctuations and periodically low water levels [2].

Since microorganisms play a key role in the lake’s biogeochemical, ecological processes and respond quickly to environmental disturbances, the exploration of their diversity is essential. Due to the touristic significance, changing trophic and water levels of the lake, numerous ecological researches were carried out, but none of them was focused on both benthic and planktonic microbial variabilities at the same time.

Our first hypothesis was that there is a link between the nutrient level and both planktonic and benthic microbial communities of the examined shallow lake. We also presumed that the planktonic and benthic microbial community of the main inflow (Zala River) is remarkedly differ from the lake and that there is an observable difference across the trophic gradient moving away from the nutrient-rich inlet (from west to the east) or among the coastal and the pelagic samples. Our last hypothesis was that the spatial heterogeneity of planktonic and benthic bacterial communities is remarkedly differ from each other. Our hypotheses were chosen to give answers for urgent questions on a sensitive shallow lake facing challenges like climate change and to support actions for maintaining the oligotrophic state and ensuring good water quality.

## Materials and Methods

### Sampling Methods and Measurement of Field Parameters

**Fig. 1:**
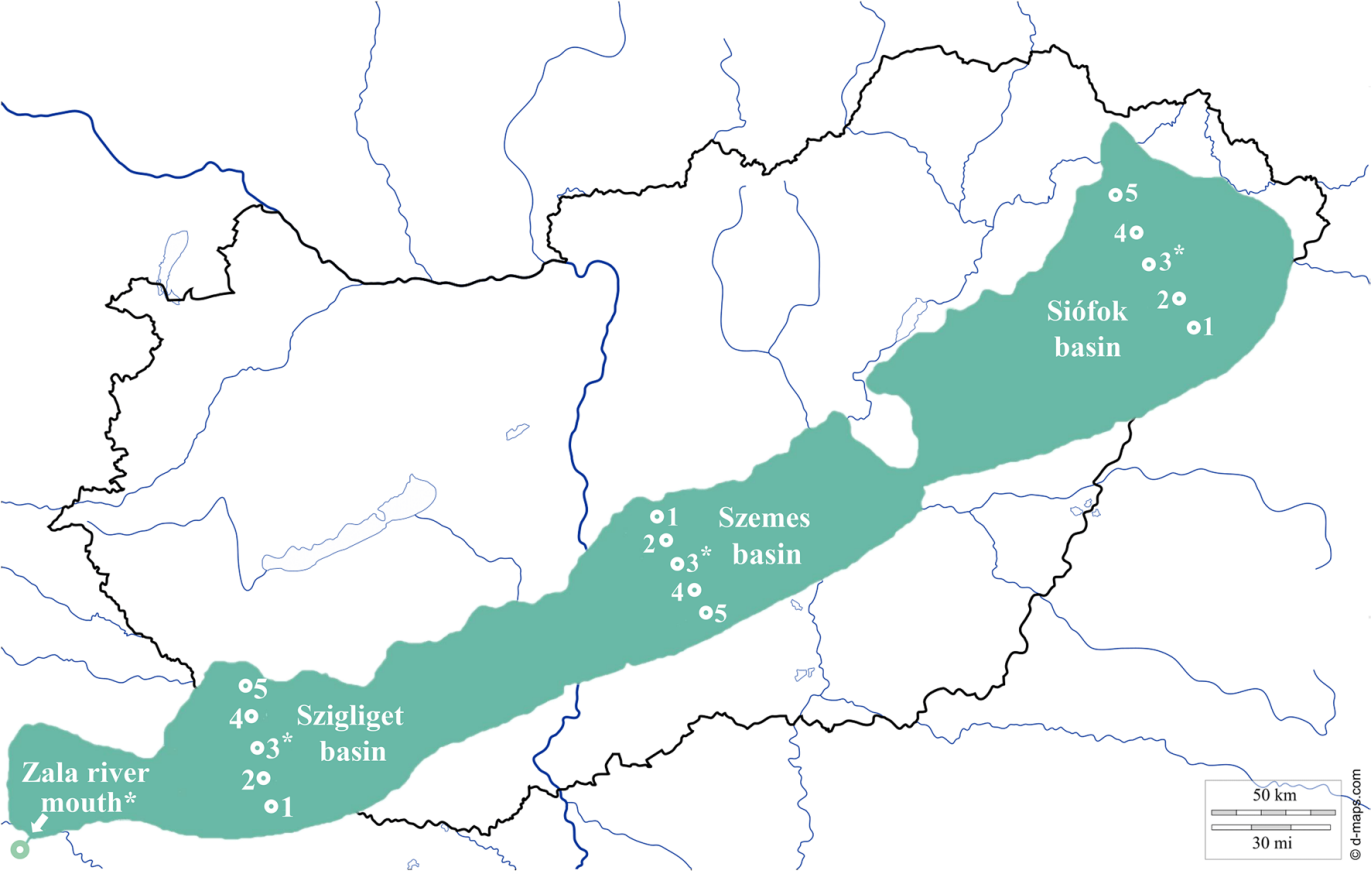
Location of the sampling site in Hungary, and the position of 16 sampling areas on Lake Balaton and the inflow Zala River (map of Hungary: https://d-maps.com/carte.php?num_car=3563&lang=en)

To reveal the spatial heterogeneity of planktonic and benthic bacterial communities, 16 sampling areas were marked out on Lake Balaton and the inflow Zala River. On 27th of June 2017, five–five cross-section composite water samples were collected into 1L sterile bottles in the Siófok-, Szemes- and Szigliget basins of the lake and one additionally at the Zala River mouth (Fig. 1). Four chosen sampling points were checked for water chemistry parameters, too, marked with an asterisk in Fig. 1. The sampling strategy was based on the international standard ISO 5667-4:2016 using three parallel transects crossing the basins from inlet to outlet designed for long-term multifunctional monitoring on the chosen shallow lake as it was suggested [3]. At the time of the sampling, following the summer operating practices, effluent water from the Kis-Balaton Water Protection System was highly retained, hence, the water inflow to the lake was only 2.2 m^3^/s. The effect of the natural sediment perturbation on the lake was marginal since the wind flow velocity was lower than 5 m/s during the day. Sediment cores from the same area were taken with an improved *Ekman-Birge* bottom mud sampler. After homogenization, the sediment samples were distributed into 200 mL sterile, brown-coloured glass bottles. Both water and sediment samples were cooled until laboratory processing. The physical and chemical parameters of the water (temperature, pH, conductivity, dissolved oxygen) were measured on-site using a HANNA HI9828 instrument (HANNA Instruments®, USA).

### Analytical Methods

Analytical methods followed the protocol described previously [4] and are shortly summarized below.

The total suspended solids (TSS) content was measured gravimetrically. Water samples (10–150 mL) were filtered through a pre-dried and pre-weighed GF-5 glass fiber filter (Whatman; pore size: 0.4 μm), were dried at 105 °C for 2 h and were reweighed. The TSS content was calculated based on the volume of the filtered sample. The CDOM content was expressed as platinum units (mg Pt/L). Water samples were filtered with a 0.45-μm pore size cellulose acetate filter and were buffered with borate buffer (pH = 8.0). Measurement of sample absorbance was performed at 440 and 750 nm wavelengths using a Shimadzu 160A UV–Vis (Japan) spectrophotometer with buffered Milli-Q ultrapure water as a blank. CDOM content was calculated as it was described previously [5]. For the measurement of DOC concentration, water samples were filtered through a pre-combusted GF-5 filter. Samples for TOC and DOC analysis were acidified to pH 2.0 using HCl and bubbled with air to remove dissolved inorganic carbon (3 replicates). TOC and DOC were measured with a High-TOC analyser (Elementar Analysensysteme, Hanau, Germany). The total nitrogen (TN) concentration was determined with the digestion of samples in the presence of NaOH and K_2_S_2_O_8_ (three replicates) in an autoclave at 110 °C for 2 h. Such treatment converts most nitrogen forms to nitrate. After pre-treatment, nitrate concentration was determined via reduction to nitrite using a previously described method [6].

To determine total phosphorus (TP) concentration, samples were digested in the presence of 5% K_2_S_2_O_8_ to convert all phosphorous forms to PO_4_^3−^ (three replicates). Phosphate and SRP concentration from the GF-5 filtered lake water was then determined using a stannous chloride method [7]. Chlorophyll *a* concentration was determined in freshly collected samples. Depending on the optical density of the sample, 100–1000 mL of lake water was filtered through a GF-5 filter, from which chlorophyll *a* was subsequently extracted using a hot methanol method. Chl *a* concentration was determined spectrophotometrically [8].

### DNA Isolation and Community Fingerprinting

Total community DNA samples were extracted from 500 mL water and 250 mg sediment samples using the DNeasy PowerSoil Kit (QIAGEN, Venlo, Netherlands), according to the manufacturer’s instructions.

The spatial heterogeneity of the bacterial community was investigated by the previously described, slightly modified terminal restriction fragment length polymorphism (T-RFLP) fingerprinting method [9]. In brief, the amplification of 16S ribosomal RNA (rRNA) T-RFLP analysis was carried out using the bacterial primers fluorescently labelled (VIC)27F (5′-AGA GTT TGA TCM TGG CTC AG-3′) and non-labelled 1492R (5′-GGT TAC CTT GTT ACG ACT T-3′). Amplification was performed in a ProFlex PCR System (Life Technologies, Carlsbad, USA) with cycling conditions as follows: 3 min initial denaturation at 95 °C, 32 cycles of amplification (30 s at 95 °C, 30 s at 52 °C, 70 s at 72 °C), and 7 min terminal extension at 72 °C. PCR mixture contained 5 μL DreamTaq Green buffer, 1 U of DreamTaq Green DNA Polymerase, 0.2 mM dNTP (Thermo Fisher Scientific, Waltham, USA), 0.3 μM of forward and reverse primers, 1 μL of template DNA, and molecular-grade water to a final volume of 25 μL.

The VIC-labelled 16S ribosomal DNA products were checked by electrophoresis on ethidium-bromide stained 1% agarose gels. The amplicons were digested with the restriction enzyme *Alu*I (AG↓CT) (Thermo Fisher Scientific, Waltham, USA) in a ProFlex PCR System (Life Technologies, Carlsbad, USA) for 90 min at 37 °C. The 10 μL reaction mixture contained 1 μL 10 × Tango Buffer, 1 U of restriction enzyme (Thermo Fisher Scientific, Waltham, USA), 8.9 μL of the template, and ultrapure water. Samples were ethanol precipitated and resuspended in sterile ultrapure water. Purified digested DNA of 0.3–1 μL was added to 10 μL formamide and 0.2 μL Genescan Liz 1200 size standard (Thermo Fisher Scientific, Waltham, USA). Fragments were separated on a Model 3130 Genetic Analyzer (Applied Biosystems, Waltham, USA), while the primary evaluation of electropherograms was performed using GeneMapper 4.0 software (Applied Biosystems, Waltham, USA). Terminal restriction fragment (T-RF) peaks with a peak height below 100 relative fluorescence units or with a peak abundance contribution below 1% were excluded from further analysis. For consensus profiles, duplicate electropherograms from each sample were aligned with each other using the T-Align program [10] at a 0.5-bp confidence interval. PAST 3.26 software [11] was used to compare the microbial community structures by the UPGMA method based on the normalized T-RFLP matrix and Bray–Curtis similarity. Diversity calculations based on the determination of Shannon–Weaver diversity index (H’) for each T-RFLP electropherogram. According to the results, 4–4 water and sediment samples were chosen for 16S rDNA amplicon sequencing.

### Illumina 16S rDNA Amplicon Sequencing and Bioinformatics Analysis

Illumina 16S rDNA amplicon sequencing was used to precisely assess the bacterial community composition of the chosen samples. For paired-end 16S rDNA amplicon sequencing, the variable V3 and V4 regions of the 16S rRNA gene were amplified using forward (5′-TCGT CGGCAGCGTCAGATGTG TATAAGAGACAGCCTA CGGGNGGCWGCAG-3′) and reverse (5′-GTCT CGTGGGCT CGGAGATGTGTATAAGAGAC AGGACTACHVGGGTATCTAATCC-3′) primers with Illumina adapter overhanging oligonucleotides [12]. PCR reaction was contained 12.5 ng of DNA, 0.2 μM of each Illumina 16S primers and 12.5 μL of 2X KAPA HiFi HotStart Ready Mix (KAPABiosystems, London, UK) supplemented with molecular-grade water to 25 μL final volume. The temperature profile was the following: initial denaturation for 5 min at 95 °C, 25 cycles of amplification (30 s at 95 °C, 30 s at 55 °C, 30 s at 72 °C). The last step was a final extension for 5 min at 72 °C. All amplifications were carried out in a ProFlex PCR System (Life Technologies, Carlsbad, USA). Amplicons were analysed by agarose gel electrophoresis. Paired-end fragment reads were generated on an Illumina MiSeq sequencer using MiSeq Reagent Kit v3 (600-cycle). Read numbers ranged between 68,526 and 94,833. Primary data analysis (base-calling) was carried out with Bbcl2fastq^ software (v2.17.1.14, Illumina). Reads were quality- and length-trimmed in CLC Genomics Workbench Tool 9.5.1 using an error probability of 0.05 (Q13) and a minimum length of 50 nucleotides as the threshold. Trimmed sequences were processed using mothur v 1.41.1 [13] as recommended by the MiSeq SOP page (https://www.mothur.org/wiki/MiSeq_SOP) [14]. The sequence assortment based on the alignment with the SILVA 132 SSURef NR99 database [15]. Chimera detection was performed with the mothur’s uchime command [16]. The ‘split.abund’ command was used to remove singleton reads [17]. The standard 97% similarity threshold was used to determine operational taxonomic units (OTUs) as it was suggested for prokaryotic species delineation [18]. Venn diagrams were generated by the mothur’s venn command. Raw sequence reads were deposited in NCBI SRA under BioProject ID PRJNA601652. *Rarefaction curves* (Fig. S1) showed high *sequencing coverage* in all samples. The most abundant OTUs were also identified using the EzBioCloud 16S rDNA database [19].

Correlation between the 10 most abundant OTUs of planktonic samples (revealed by next-generation sequencing), environmental variables (TSS, DOC, TOC, TN, TP, SRP, chlorophyll, humic substances), and sampling areas (Siófok Basin, Szemes Basin, Szigliget Basin, and Zala River) was calculated with canonical correspondence analysis (CCA) using PAST 3.26 software [11].

## Results

### Limnological Results

Measured physical and chemical parameters reflected typical early summer values of the lake (Table 1). A phosphorus gradient was observable from west to the east, which is an expected outcome of the Zala River contribution to the nutrient load of the lake, while the distribution of nitrogen and carbon was not so specific at the time of sampling. A similar phosphorus gradient was also detected earlier in the interstitial water of the sediments [20].

**Table 1:** Physical and chemical parameters of water samples in June 2017

Sampling area	Siófok basin	Szemes basin	Szigliget basin	Zala River
GPS coordinate	46° 59′ 09.4′′ N	46° 50′ 39.7′′ N	46° 45′ 06.7′′ N	46° 42′ 06.7′′ N
	18° 04′ 44.8′′ E	17° 44′ 35.7′′ E	17° 25′ 08.7′′ E	17° 15′ 30.7′′ E
Water temperature (°C)	25.6	26.2	25.8	25.0
pH	8.7	8.6	8.6	8.4
Dissolved oxygen (mg/L)	8.1	8.0	8.7	4.1
Conductivity (µS/cm)	790	755	751	831
ORP (mV)	206	193	226	126
Chlorophyll (µg/L)	2.5	3.2	5.9	25.4
CDOM (mg Pt/L)	4.0	5.7	14.1	103.0
T.S.S (mg/L)	4.7	7.7	11.5	4.7
SRP (µg/L)	3.0	4.3	5.6	96.6
TP (µg/L)	18	20	31	179
TN (µg/L)	914	858	895	1674
DOC (mg/L)	8.9	9.1	9.0	19.7
TOC (mg/L)	9.9	9.3	10.2	20.4

At the time of our investigation, based on chlorophyll (2.5–5.9 µg/L) and phosphorus (18–31 µg/L) values, Balaton could be classified into a mesotrophic state. In the western basin, depending on water temperature and the duration of sunlight, chlorophyll generally increases up to 30–60 µg/L average value during the late summer and early autumn. Accordingly, the lake can reach eutrophic status by late summer.

### 16S rDNA T-RFLP Profiles of Planktonic and Benthic Samples

**Fig. 2:**
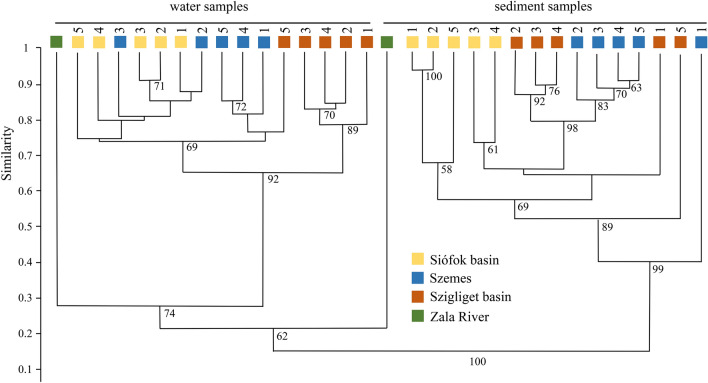
UPGMA dendrogram of 16S rDNA T-RFLP fingerprints of planktonic and benthic bacterial communities based on Bray–Curtis similarity index. The different sampling sites are indicated with different colour and number

Results of the T-RFLP fingerprinting are shown in the UPGMA dendrogram (Fig. 2). The Shannon–Weaver indexes of the water samples of the entire eastern basin and in the middle of the Szemes basin were low (2.24–2.48), while samples of the near-coastal areas and the western basin showed higher diversity values (2.58–2.78). The diversity index of the sediment samples ranged between 2.60 and 3.27, but there was no observable difference across the trophic gradient neither from west to the east nor among the coastal and the pelagic samples. T-RFLP fingerprints of the low diversity water samples (Siófok basin, and Szemes basin 2–3) clustered together with a similarity value of ~ 75%. The remaining water samples formed two distinct clusters (Szigliget basin 1–4 and the rest of the samples). Among all, Zala River 16S rDNA fingerprints had the most unique profiles, since both planktonic and benthic samples considerably differed from the lake ones. Most of the lake sediment samples were composed of soft, grey-coloured, calcite-rich colloids. Still, samples from different basins formed significantly distinct groups. However, in the case of the Szemes 1 sediment sample, we found a large amount of heavier particles dominated by quartz-rich clastic deposits (due to its near-shore location and the wave breaking effect), which observation explained its outlying nature at the UPGMA dendrogram. The Szigliget 5 sediment sample was also distant from the others since it is originated close to the estuarine area of Lesence-, Kétöles- and Tapolca streams delivering a significant amount of organic material into the lake.

To uncover the bacterial diversity, 4–4 water, and sediment samples were chosen for 16S rDNA amplicon sequencing originating from the Zala River and the middle sampling points of the Siófok-, Szemes- and Szigliget basins (marked with no. 3 on Fig. 1).

### Illumina 16S rDNA Amplicon Sequencing Results

The Shannon–Weaver diversity indexes calculated by the 16S amplicon sequencing data showed the same distribution as the T-RFLP indicated. The OTU cluster analysis revealed that the eight samples were clustered into four different groups. The lake sediment and water samples formed two distinct clusters, while Zala River samples had more unique community profiles (Fig. S2).

**Fig. 3:**
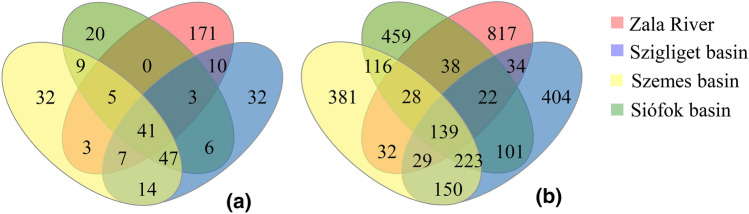
The number of exclusive and shared bacterial OTUs identified by 16S Illumina amplicon sequencing retrieved from water (**a**) and sediment (**b**) samples

Diversity indexes of the planktonic samples were the lowest at Siófok basin sampling points and increased along the trophic gradient to Zala River (3.6–3.76–3.9–4.1), whereas diversity indexes of benthic communities were almost equal at the four sampling sites (5.74–5.84). Venn diagrams were generated to compare the similar and different OTUs among the water and sediment samples, respectively (Fig. 3). In the case of planktonic communities, the number of sampling point-specific OTUs ranged from 20 (Siófok basin) to 171 (Zala River) and the rate of shared OTUs was more than 10% (41 from the total 399). At the same time, the number of site-specific OTUs of benthic microbial communities was higher (381–817) and the rate of shared OTUs was less significant (139 from the total 3013).

### Microbial Diversity of the Planktonic Samples

**Fig. 4:**
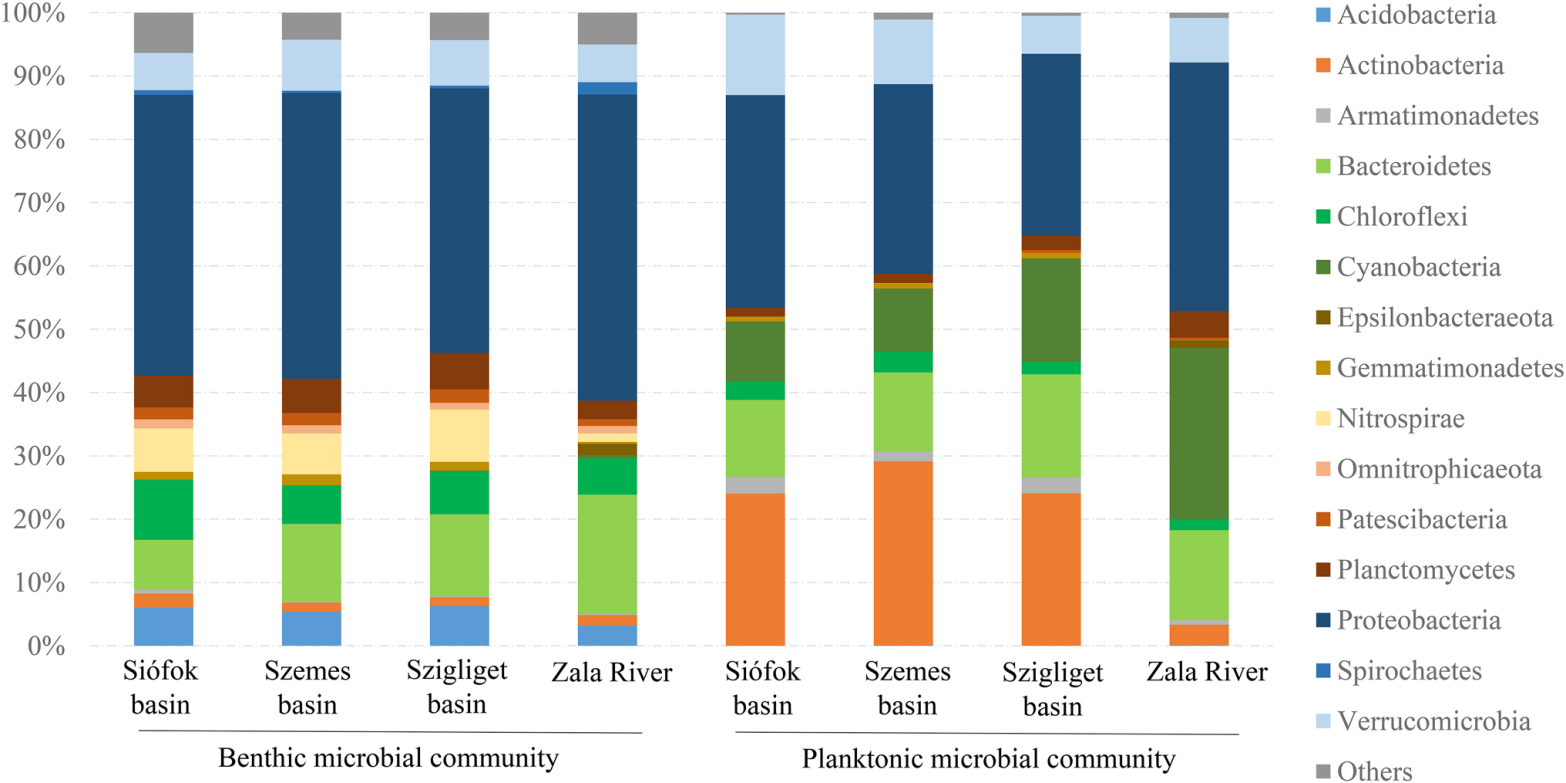
Phylum level distribution obtained by Illumina 16S rRNA gene amplicon sequencing of benthic and planktonic microbial communities of Lake Balaton and the main inflow Zala River

Bacterial communities of the water samples were dominated by members of Proteobacteria (28–39%), Bacteroidetes (12–16%), Cyanobacteria (10–29%), and Verrucomicrobia (7–12%). Actinobacterial lineages (24–29%) were abundant only in the Lake Balaton samples (Fig. 4).

**Fig. 5:**
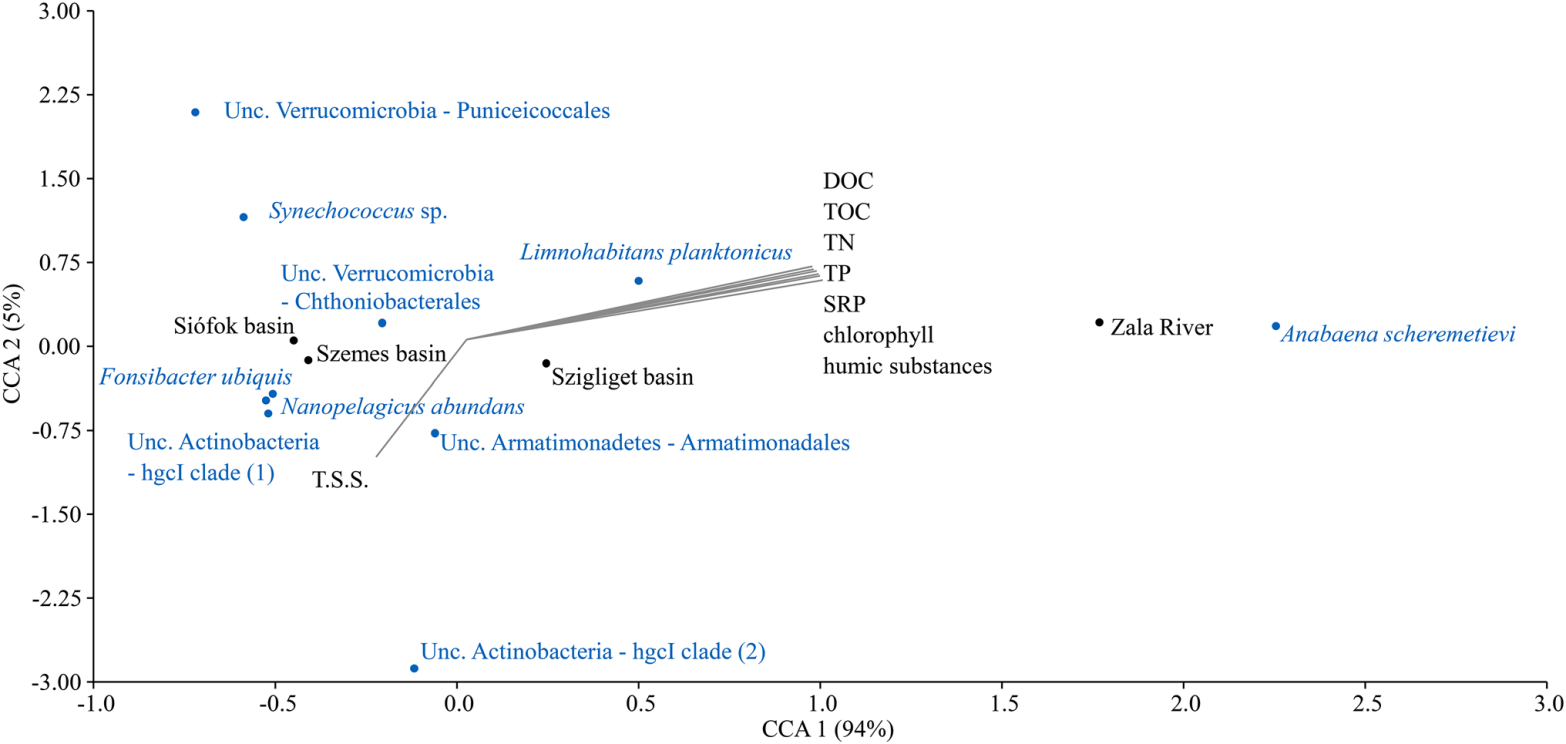
Canonical Correlation Analysis (CCA) between 10 most abundant microbial OTUs of planktonic samples, environmental factors and sampling areas

Within the Proteobacteria phylum, a different composition could be observed among the samples: the ratio of Alpha- and Gammaproteobacteria were nearly equal (between 18 and 13%) in the case of the lacustrine samples, while mainly the latter class dominated (32%) the riverine one. Most of the alphaproteobacterial sequences belonged to the freshwater LD12 subclade member *Fonsibacter ubiquis* (Table S1). We have noticed a slightly decreasing abundance of this lineage along the trophic gradient (15% in the eastern basin, 13% in the middle- and 11% in the western basin), and the result of the canonical correlation analysis (CCA) also suggested that this genus prefers lower nutrient density (Fig. 5).

The gammaproteobacterial sequences belonged to several genera of the Betaproteobacteriales. Among all, the typical planktonic freshwater genus *Limnohabitans* was the most abundant (3–5%) in all samples. The dominant genotype showed 99.1% 16 s rDNA sequence similarity to *Limnohabitans planktonicus* II-D5^T^. At the Zala River, unknown members of Methylococcales were highly represented (10%), and an unknown *Azovibrio*-related bacterium was also present in a notable amount (5%).

Within the Bacteroidetes phylum, several genera (e.g. *Flavobacterium, Fluviicola*, *Candidatus* Aquirestis, *Terrimonas*, *Dinghuibacter, NS11-12 marine group*) were detected in a low abundance (1–3%). The *Flavobacterium* genus represented less than 0.5% of the lake planktonic diversity, while in the river sample it was more abundant (2.5%). The *NS11-12 marine group* and the *Fluviicola* species showed a more homogenous distribution; however, the latter bacteria were more frequent in the estuarine area. Members of the genera *Terrimonas*, *Candidatus* Aquirestis, and *Dinghuibacter* were only abundant in the lacustrine samples.

The number of primary producer Cyanobacteria was higher in the nutrient-dense River Zala (27%) and in the western basin of the lake (16%), which is close to the estuarine area. In the riverine sample, this phylum was dominated by a member of the *Anabaena* genus (*A. scheremetievi* with a 99.8% 16S rDNA sequence similarity), while in the Szigliget basin, the also filament-forming *Aphanizomenon flos-aquae* was the most abundant. According to the CCA plot, *A. scheremetievi* showed a positive correlation with the growing quantity of nutrients (Fig. 3). Members of the genus *Microcystis* were also found in a notable rate (2.8%) in the Zala River, but not in the lake. In the relatively nutrient-poor middle and eastern basins of the lake, the abundance of picoplanktonic members of Cyanobacteria was higher (8–9%) than in the nutrient-rich Szigliget basin (4%). The dominant sequences showed 99% pairwise similarity to the phycocyanin producing *Synechococcus* sp. MA0607K, which was previously isolated from the Great Mazurian Lakes system [21].

The abundance of the ubiquitous Verrucomicrobia bacteria increased from the estuarine area to the east (from 6 to 12%). The two most abundant OTUs belonged to the uncultured members of Puniceicoccales, and Chthoniobacterales. According to the spatial distribution of these bacteria, they may prefer more oligotrophic conditions.

The actinobacterial sequences showed a wide variety, although most of them affiliated with the hgcI clade (also known as acI clade), such as *Nanopelagicus abundans* (7–8%), *Planktophila limnetica* (~ 2%), and two, yet uncultured microorganisms (1.3 and 3.1%, respectively). A considerable quantity (1.8–2.6%) of unknown Ilumatobacteraceae bacteria (CL500-29 marine group) was also detectable in the lacustrine samples.

### Microbial Diversity of the Benthic Samples

The benthic samples were dominated by members of Proteobacteria (40–45%), Bacteroidetes (7–18%), Chloroflexi (6–9%), Verrucomicrobia (6–9%), Nitrospirae (1–8%), Planctomycetes (4–6%), and Acidobacteria (3–6%).

Within the Proteobacteria phylum, Gammaproteobacteria were highly dominant (23–30%) and an unknown Steroidobacterales bacterium was the most abundant in all samples (2.3–3.5%). Sequence reads recovered in the present study showed a 99.6% 16S rRNA gene similarity to clones detected in the sediment of shallow hypereutrophic lakes in Japan [22] and China [23]. The closest cultivated relative (*Steroidobacter agariperforans*) shares only a 93.5% 16S rDNA similarity with sequences presented in our work (Table S2).

In the Zala River, the family of Rhodocyclaceae was the most dominant gammaproteobacterial group (6%), represented mainly by *Dechloromonas denitrificans*-related bacteria (with a 98.8% 16S rRNA gene homology). The second abundant group was the type I methanotroph family Methylococcaceae (4.5%) (mainly methane-oxidizing Methylobacter tundripaludum, 97.8%). An unknown Thiobacillus (with a 97.8% similarity to *Thiobacillus denitrificans*) was also abundant in the estuary and the eastern basin of the lake. The members of the family Nitrosomonadaceae were found primarily (3–3.6%) in the lake samples, but not in the riverine sample.

The abundance of Alphaproteobacteria was ten times less (~ 2%) in the benthic samples than in the planktonic ones, while the abundance of Deltaproteobacteria (12–18%) showed an increased level. In the lake sediment samples, the most abundant deltaproteobacterium (2–3%) was an unknown Desulfarculaceae species (with only 94% similarity to Desulfatiglans aniline). The related sequence reads showed a 99.6% similarity to 16S rDNA clones detected in the sediment of the aforementioned shallow hypereutrophic lake in Japan [22] and another shallow lake in China [24]. An unknown Desulfobacteraceae bacterium was detectable in all the samples (~ 2%), while Geobacteraceae-related bacteria were only notable in the River Zala.

Bacteroidetes were abundant in the river sediment (18%) and decreased alongside the trophic gradient (13–12–8%, respectively). Within the phylum, more than 600 OTUs were detectable but only an unknown Ignavibacteriaceae (closest relative *Ignavibacterium album* with a 93.6% similarity) was abundant in the lake sediment samples (1.5–2.5%). The Chloroflexi- and Verrucomicrobia-related OTUs also showed a great diversity: in the first phylum, unknown members of the methanogenic Anaerolineaceae family (3–3.7%), while in the latter, unknown members of Pedosphaeraceae family were detectable in a significant amount. Most of the Nitrospira-related reads showed 98.5% similarity with *Nitrospira nitrificans*, a species which’s genome encodes all enzymes necessary for ammonia oxidation via nitrite to nitrate [25]. The abundance of this group was low in the river (0.06%) and increased from east to west (1–1.8–3.2%) in the lake sediments.

Planctomycetes phylum was more abundant in benthic (4–6%) than in planktonic samples (1–2%). Sequences showed a wide variety (681 different OTUs) and most of them belonged to the Pirellulaceae family.

## Discussion

By the time of writing, only a few studies have investigated the planktonic and benthic microbial diversities of the largest central European shallow lake and its main inflow. So far, studies have mainly focused on the picophytoplankton members of the microbial community or relied on culturing methods and 16S DGGE fingerprinting [20, 26]. To the best of our knowledge, the present study provides the first comprehensive analysis of the planktonic and benthic bacterial communities of the Lake Balaton and the main inflow Zala River. Based on molecular biological (16S rDNA T-RFLP fingerprinting and amplicon sequencing) analysis, the planktonic and benthic microbial communities of the lake formed two distinct clusters, while Zala River samples have more individual profiles (Figs. 2, S2) which is supporting our initial hypotheses.

The planktonic microbial communities were dominated by Proteobacteria, Bacteroidetes, Cyanobacteria, and Verrucomicrobia in all samples. At the same time, the Actinobacterial lineages were less abundant in the water body of the river, similar to a previous study on the abundance of actinobacteria in Danish streams and fish ponds [27]. Within Proteobacteria, the ratio of Alpha- and Gammaproteobacteria were nearly equal among the lacustrine samples, while in the riverine sample mainly members of the latter class were found. The alphaproteobacterial sequences primarily belonged to the LD12 subclade member *F. ubiquis*. This bacterium was a frequent bacterial community member (up to 25% abundance) in several shallow and deep lakes, but only low proportions of LD12 bacteria were detectable in deep waters [28]. *F. ubiquis* was also abundant in Lake Balaton in 2013 summer [29]. This small oligotrophic bacterium usually feeds on dissolved organic carbon and nitrogen and seems to prefer relatively high pH and low DOC/TP values [30]. Our observation also suggests that this bacterium prefers lower nutrient density, since the abundance of this lineage was slightly decreasing along the trophic gradient.

The Gammaproteobacteria-related bacteria were revealed in a wide variety. Members of the Betaproteobacteriales showed the highest diversity, among which *L. planktonicus* was the most abundant. This bacterium, along with *L. parvus,* belongs to the R-BT lineage which may give up to 30% of free-living bacteria in a broad range of shallow freshwater habitats including nonacidic lakes, ponds, and rivers [31, 32]. According to previous findings [33], these R-BT lineage members may play a key role in carbon flow from algal-derived substrates to the plankton grazers. We also found a positive correlation between the presence of this species and the chlorophyll values (Fig. 5).

In anoxic sediments, a significant amount of methane can be released by methanogenic archaea, which is consecutively being oxidized in the water column and on the surface of the sediment, mainly by aerobic alpha‐ or gammaproteobacterial methane‐oxidizing bacteria [34]. This process was mediated by the latter class in the planktonic community of Zala River since unknown members of Methylococcales were highly abundant (10%). The presence of methanotrophic Gammaproteobacteria can be a result of benthic-pelagic exchange due to sediment re-suspension as it was previously described in the oligotrophic Lake Constance [35]. Unfortunately, the used primer set is not able to amplify the archaeal sequences, and the concentration of methane was not investigated, so this presumption needs further investigation.

Planktonic bacteria belonging to the phylum Bacteroidetes also showed a high versatility since several genera (e.g. Flavobacterium, Fluviicola, Candidatus Aquirestis, Terrimonas, Dinghuibacter, NS11-12 marine group) were found in a low abundance (1–3%). Most of them were previously detected in other Hungarian shallow lakes [36, 37]. The massive presence of the mostly chemoorganotrophic Bacteroidetes-related bacteria in shallow freshwater habitats is often related to the high level of dissolved organic carbon concentration derived by algal blooms or from external inputs [38, 39]. Since in Lake Balaton the chlorophyll and the DOC values were relatively low at the time of sampling (early summer) (Table 1), the low rate of the above-mentioned genera is understandable.

The primary producer Cyanobacteria was simultaneously abundant in the nutrient-dense River Zala (27%) and the western basin of the lake (16%). In these areas, the phylum was dominated by members of the filament-forming *Anabaena* and *A. flos-aquae* species. Interestingly *Anabaena* genus was found mainly in the estuarine area while *A. flos-aquae* species were exclusively observed in the Szigliget basin (Table S1). *A. scheremetievi,* the most dominant species showed a positive correlation with the increasing level of nutrients (Fig. 5). The presence of these Cyanobacteria corresponds to an earlier finding on the seasonal pattern of the algal community in Lake Balaton. According to former studies, during the spring period (with low temperature and high light intensity) *A. flos-aquae* becomes dominant, while during the summer period, the self-shading effect of the increased algal biomass is favourable for other heterocytic species (e.g. *A. issatschenkoi* and *Anabaena*) with a lower light requirement. In late summer and early autumn, *Cylindrospermopsis raciborskii* becomes dominant since their lower light requirement is an advantage over other N_2_-fixing cyanobacteria [40].

*Microcystis* species of Cyanobacteria were detected in a significant proportion (2.8%) in the Zala River only. In the ‘60 s, as a result of high external nutrient load, the members of this genus (*Microcystis aeruginosa and M. flos-aquae*), together with the *A. flos-aquae,* frequently caused cyanobacterial blooms in the Lake Balaton. Since then, strict water quality regulations have been introduced, therefore only one local blooming of *Microcystis flos-aquae* was observed along the northern coastline of the eastern basin in 2015 [41]. Our findings are verifying the long-term effect and the success of these regulations.

In the frame of our work, the picoplanktonic members of Cyanobacteria were also detected. Due to their high surface-area-to-volume ratio, these bacteria usually prefer meso- and oligotrophic conditions [42]. Thus, with the decrease of trophy towards the Siófok Basin, the role of *Synechococcus* picoalgae in the total production increased (from 4% in the nutrient-dense Szigliget basin to 8–9% of the sequence reads in the middle and eastern basins). Our results are consistent with previous measurements on the lake performed in 2006, which showed that in Lake Balaton, like in other lakes, there is an inverse relationship between the proportion of picoalgae and the nutrient density of the water column [43]. A previous population dynamics study [44] revealed that the *Synechococcus* genus showed a growing period in spring and had one or two abundance peaks until autumn. In Lake Balaton, the high abundance of *Synechococcus*-related picocyanobacteria was demonstrated during summer [26].

The number of ubiquitous Verrucomicrobia bacteria increased from the estuarine area to the east (from 6 to 12%), like picoalgae. Despite the high relative abundance (2–20%) found worldwide in freshwater ecosystems, the role of these bacteria remains unclear. According to a few studies based on Verrucomicrobia *metagenome*-assembled and single-cell amplified *genomes*, this clade contains a wide variety of microbes. Small, general heterotrophs and large copiotrophs, with polysaccharide degrading pathways, and some with special features such as green-light absorbing rhodopsins and nitrogen-fixing gene sets were found among them [45]. The particle-associated verrucomicrobial communities appeared to be primarily influenced by phytoplankton richness, rotifer abundance, and inorganic nutrients, whereas the free-living fraction was correlated with the biomass dynamics of some phytoplankton classes (Chlorophyceae, Chrysophyceae, Desmidiaceae, and Zygnemataceae) [46].

Most of the actinobacterial sequences affiliated with the hgcI clade (also known as acI clade) member *N. abundans*, *P. limnetica,* and two yet unknown microorganisms. A considerable quantity of unknown Ilumatobacteraceae bacteria (CL500-29 marine group) was also detectable. The actinobacterial hgcI clade was found to be common in a wide range of freshwater habitats, and it could account for > 50% of planktonic microbial communities in lakes [47]. The clade members usually have extremely small cell and genome size that has been explained by metabolic dependency between this type of co-occurring, free-living bacteria. The ‘Ca. Nanopelagicales’ have been found mostly in the epilimnion, showing seasonal dynamics that correlate to algal, picocyanobacterial blooms, and the high occurrence of heterotrophic nanoflagellates [48]. ‘Ca. P. limnetica’ is also a frequent planktonic representative of fresh- and possibly slightly oligosaline inland waters [49]. Both microorganisms possess actinorhodopsins, which can generate energy from the green light and this photoheterotrophic metabolism helps them to survive in the oligotrophic environment. The higher organic matter concentration might have negative effects on the abundance of acI clade and CL500-29 marine group members [50]. Moreover, these bacteria were more abundant in the less nutrient-dense pelagic zone in the second largest shallow lake of Hungary, Lake Fertő [37]. In our case, the presence of three hgcI clade members was also higher in the relatively nutrient-poor middle and eastern basins but none of them showed a characteristic distribution. Interestingly one yet unknown clade member was abundant in the western basin and decreased alongside the trophic gradient.

In the benthic samples, microorganisms adapted to less aerated deeper layers were observable. Within Proteobacteria, the Gammaproteobacteria class was highly abundant (23–30%). At the species level, an unknown Steroidobacteraceae bacterium was the most frequent in all samples, which was previously detected in the sediment of two Hungarian shallow soda pans, situated at a nature conservation area [51]. The presence of Methylococcales was high in the planktonic samples and similarly, it was remarkable in the sediments. However, the abundance of this family was only high in the estuarine area (4.5%), while in the lacustrine samples only a low amount of related species was observable (0.2–0.5%). This finding also supports our hypothesis, that methanogenic archaea should be abundant members of the riverine benthic microbial community. In the River Zala sediment, Rhodocyclaceae species were also detected in a large number, and the sequences were closely related to the facultative anaerobe denitrifier *D. denitrificans*. The members of this family are typical in microbial communities of freshwater sediments [52].

The aerobic ammonia oxidizer Nitrosomonadaceae family were found primarily (3–3.6%) in the lake samples, but not in the riverine sample. Interestingly, the distribution of the ammonia oxidizer Nitrospira genus was quite similar. Moreover, the other well-known ammonia-oxidizing genera (Nitrosolobus and Nitrosovibrio) were also absent in the riverine samples. Because of that, the ammonia oxidation in the benthic region of River Zala should be carried out by archaeal species.

Due to the presence of diverse sulphate-reducing bacteria (~ 6% in all samples), the abundance of Deltaproteobacteria (12–18%) in benthic environments was higher than in planktonic samples. Desulfarculales were abundant in lacustrine and Desulfuromonadales in riverine samples, while Desulfobacteraceae were frequent in both areas.

The abundance of Bacteroidetes-related bacteria was the highest in the river sediment and decreased alongside the trophic gradient. Within the phylum, more than 600 OTUs were detectable, but only an unknown Ignavibacteriaceae was abundant. Its 16S rDNA sequence showed 93.8% similarity with the only valid species, *I. album*, of the genus. According to the low similarity, the environmental role of this species in our samples is unclear. The Chloroflexi- and Verrucomicrobia-related OTUs also showed great diversity. However, in the first phylum, unknown members of the methanogenic Anaerolineaceae family (3–3.7%), while in the latter, unknown members of the Pedosphaeraceae family were detectable in a significant amount. These two families were widely distributed in the benthic communities of a large shallow eutrophic lake in China [53].

Planctomycetes phylum was more frequent in sediment (4–6%) than in water samples (1–2%), since these bacteria prefer a surface-attached lifestyle. Study of four lakes in north-eastern Germany, showed a complete absence of Planctomycetes was found among the free-living microorganisms, while the group appeared abundant among the sediment surface colonizers [54].

## Conclusion

In Lake Balaton, a phosphorus gradient was observable from west to the east, which is a verification of our hypothesis and an expected outcome of the Zala River contribution to the nutrient load of the lake. At the time of sampling, the distribution of nitrogen and carbon was not so specific. The effect of the main inflow Zala River on the composition of the microbial community during the study period was even less significant since the lacustrine and the riverine samples had markedly different profiles. Only a slight difference was observable across the trophic gradient moving away from the nutrient-rich inlet (from west to the east) or among the coastal and the pelagic samples. The marginal impact of the river may be explained by the summer operating practice, namely that the water of Zala River was intensively retrained, so the inflow discharge to the lake was low. Nevertheless, the difference could be a result of the higher level of nutrients as well.

The planktonic microbial community was mainly dominated by species detected in shallow waters. According to the molecular biological results, mostly well-known freshwater microorganisms, adapted to nutrient-poor conditions (Verrucomicrobia, hgcI clade members of Actinobacteria, LD12 subclade members of Alphaproteobacteria) were found in the pelagic water column. Eutrophic conditions preferring filamentous cyanobacteria species were also detectable in the estuary (*Anabaena* sp.) and the western part of the lake (*A. flos-aquae*). The benthic microbial community showed higher diversity, and the appearance of microorganisms adapted to less aerated deeper layers was also observable. Members of the sulphate-reducing Desulfarculales, Desulfobacterales and Syntrophobacterales, the nitrite-oxidizing Nitrospira genus, and the surface colonizer, mainly aerobe Planctomycetes phylum dominated these samples. Our findings on the composition of planktonic and benthic microbial communities support our last hypothesis since they are remarkably different from each other.

Our study confirmed, that (i) the LD12 subclade member *F. ubiquis* and the (ii) cyanobacterial *Synechococcus* spp. prefer low nutrient density, (iii) hgcI clade members show various distribution indicating that higher organic matter concentration does not necessarily have a negative influence on their abundance, (iv) Steroidobacteraceae can be common members in sediment microbial communities of the examined shallow freshwater lake.

## Electronic supplementary material

Below is the link to the electronic supplementary material.

Supplementary file1 (PDF 429 kb). Fig. S1 Rarefaction curve of Illumina MiSeq 16S amplicon sequencing dataset—Fig. S2 UPGMA dendrogram showing OTU cluster analysis of 16S amplicon sequencing data based on Bray-Curtis similarity

## Data Availability

Raw 16S rRNA Illumina sequence reads were deposited in NCBI SRA under BioProject ID PRJNA601652.
