# Supplementary material for: Planktonic and Benthic Bacterial Communities of the Largest Central European Shallow Lake, Lake Balaton and Its Main Inflow Zala River
Source: Curr Microbiol. 2020 Oct 17;77(12):4016–28. doi: 10.1007/s00284-020-02241-7 (PMC7677278; doi:10.1007/s00284-020-02241-7)
Supplement: Supplementary file 1 — Supplementary file1 (PDF 429 kb). Fig. S1 Rarefaction curve of Illumina MiSeq 16S amplicon sequencing dataset—Fig. S2 UPGMA dendrogram showing OTU cluster analysis of 16S amplicon sequencing data based on Bray-Curtis similarity [file 284_2020_2241_MOESM1_ESM.pdf]

Planktonic and benthic bacterial communities of the largest central European shallow lake, Lake Balaton and its main inflow Zala River

[1]Milán Farkas, [1]Edit Kaszab, [1]Júlia Radó, [1]Judit Háhn, [1]Gergő Tóth, [1]Péter Harkai, [2]Árpád Ferincz, [3]Zsófia Lovász, [4]András Táncsics, [5]Lajos Vörös, [1]Balázs Kriszt, [1]Sándor Szoboszlay

[1] Department of Environmental Protection and Safety, Szent István University, Páter Károly utca 1, H-2100 Gödöllő, Hungary

[2] Department of Aquaculture, Szent István University, Páter Károly utca 1, H-2100 Gödöllő, Hungary

[3] Department Kis-Balaton, West-transdanubian Water Directorate, Csík Ferenc sétány 4. H-8360 Keszthely, Hungary

[4] Regional University Center of Excellence in Environmental Industry, Szent István University, Páter Károly utca 1, H-2100 Gödöllő, Hungary

[5] Balaton Limnological Institute, Centre for Ecological Research, Klebelsberg Kuno utca 3, H-8237 Tihany, Hungary

Corresponding author: Milan Farkas, e-mail address: mil.farkas@gmail.com, telephone number: +3628522000/1611 ext. or +36704166128, ORCID id 0000-0002-7807-4750

Fig. S1

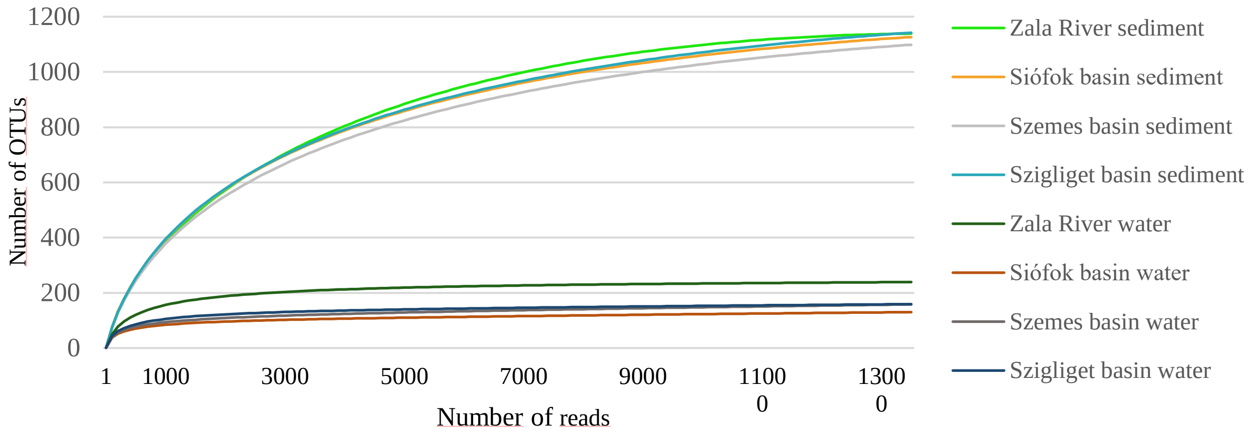

1200
1000
800
600
400
200
0
Number of OTUs
1
1000
3000
5000
7000
9000
1100
0
1300
0
Number of reads
Zala River sediment
Siófok basin sediment
Szemes basin sediment
Szigliget basin sediment
Zala River water
Siófok basin water
Szemes basin water
Szigliget basin water

Fig. S2

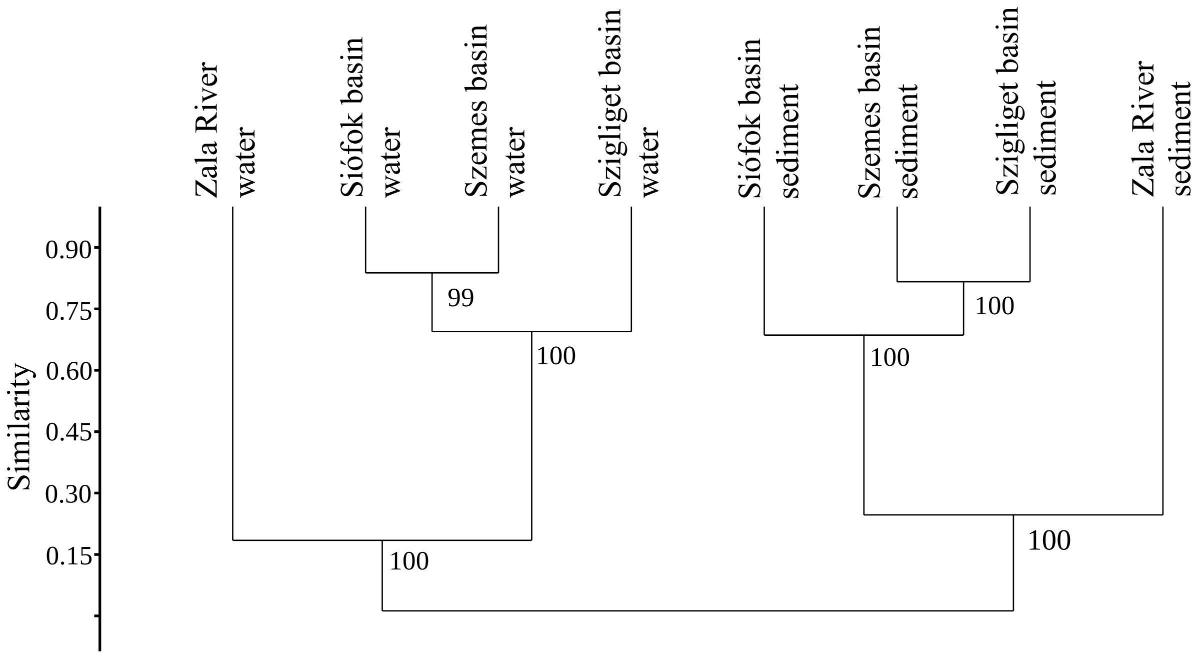

Zala River water
Siófok basin water
Szemes basin water
Szigliget basin water
Siófok basin sediment
Szemes basin sediment
Szigliget basin sediment
Zala River sediment
Similarity
0.90
0.75
0.60
0.45
0.30
0.15
99
100
100
100
100
100
100

Table S1 Identification of the top 20 planktonic microbial OTUs by the EzTaxon database database and their relative abundance at the sampling sites

Order	closest relative (EzTaxon)	% similarity	Siófok basin	Szemes basin	Szigliget basin	Zala River
Pelagibacterales	*Fonsibacter ubiquis*	99.8	15.4%	13.3%	11.2%	0.0%
Nanopelagicales (hgcI clade)	*Nanopelagicus abundans*	99.8	8.5%	8.7%	6.7%	0.1%
Chroococcales	*Synechococcus* sp. MA0607K	99.1	9.1%	8.3%	3.7%	0.8%
Nostocales	*Anabaena scheremetievi*	99.8	0.0%	0.1%	1.2%	19.5%
Puniceicoccales	*Cephaloticoccus capnophilus*	85.2	6.5%	4.4%	1.5%	0.3%
Chthoniobacterales	*Terrimicrobium sacchariphilum*	93.6	2.9%	3.5%	2.4%	1.0%
Betaproteobacteriales	*Limnohabitans planktonicus*	99.1	2.4%	1.5%	2.5%	2.7%
Frankiales (hgcI clade)	*Acidothermus cellulolyticus*	92.1	2.7%	3.0%	2.2%	0.0%
Armatimonadales	*Capsulimonas corticalis*	85.7	2.7%	1.6%	2.5%	0.7%
Nanopelagicales (hgcI clade)	*Nanopelagicus abundans*	94.8	1.4%	2.1%	3.2%	0.3%
Nostocales	*Aphanizomenon flos-aquae*	100	0%	0%	6.8%	0%
Acidimicrobiales (CL500-29 marine group)	*Ilumatobacter fluminis*	92.3	2.2%	2.5%	1.8%	0.0%
Betaproteobacteriales	*Burkholderia rinojensis*	94.2	2.6%	2.4%	1.5%	0.1%
Frankiales (hgcI clade)	*Planktophila limnetica*	99.6	2.0%	2.4%	1.6%	0.5%
Micrococcales	*Limnoluna rubra*	99.3	1.8%	2.7%	1.2%	0.7%
Chloroflexales	*Roseiflexus castenholzii*	81.8	1.7%	2.1%	1.8%	0.0%
Betaproteobacteriales	*Methylophilus leisingeri*	95.7	2.0%	1.5%	2.0%	0.0%
Chitinophagales	*Parasegetibacter terrae*	95.2	1.3%	1.6%	1.8%	0.6%
Saprospirales	*Aquirestis calciphila*	99.8	2.0%	1.3%	1.6%	0.2%
Chroococcales	*Merismopedia tenuissima*	98.6	0.3%	1.4%	1.9%	1.4%

Table S2 Identification of the top 20 benthic microbial OTUs by EzTaxon database and their relative abundance at the sampling sites

Order	closest relative (EzTaxon)	% similarity	Siófok basin	Szemes basin	Szigliget basin	Zala River
Steroidobacterales	*Steroidobacter denitrificans*	92.5	2.88%	3.69%	2.77%	2.27%
Desulfarculales	*Desulfatiglans anilini*	94.1	2.06%	3.03%	2.85%	0.64%
Ignavibacteriales	*Ignavibacterium album*	93.8	1.45%	2.37%	2.59%	0.90%
Betaproteobacteriales	*Thiobacillus denitrificans*	97.9	2.58%	0.96%	0.47%	3.14%
Nitrospirales	*Nitrospira nitrificans*	98.5	0.81%	1.84%	3.19%	0.06%
unclassified Deltaproteobacteria	*Dissulfurirhabdus thermomarina*	88.1	1.27%	2.02%	1.96%	0.03%
Nitrospirales	*Nitrospira calida*	95.8	1.18%	1.74%	1.80%	0.17%
Betaproteobacteriales	*Collimonas fungivorans*	95	0.92%	1.39%	1.38%	1.00%
Desulfobacterales	*Desulfonema magnum*	93.4	1.79%	1.31%	1.10%	0.35%
Desulfuromonadales	*Geobacter soli*	88	1.62%	1.36%	1.07%	0.07%
Nitrospirales	*Magnetobacterium bavaricum*	91.1	0.87%	1.24%	1.59%	0.04%
Anaerolineales	*Bellilinea caldifistulae*	89.1	1.22%	1.05%	1.20%	0.17%
Steroidobacterales	*Steroidobacter denitrificans*	89.1	0.51%	1.35%	1.50%	0.11%
Betaproteobacteriales	*Aquabacterium limnoticum*	96.3	0.90%	1.32%	1.10%	0.02%
Betaproteobacteriales	*Dechloromonas denitrificans*	98.7	0.00%	0.00%	0.01%	3.84%
Pedosphaerales	*Pedosphaera parvula*	91.8	0.59%	0.86%	1.18%	0.43%
Methylococcales	*Methylobacter tundripaludum*	97.8	0.01%	0.03%	0.05%	3.38%
Nevskiales	*Povalibacter uvarum*	90.8	0.45%	0.53%	0.41%	1.49%
Desulfuromonadales	*Geobacter soli*	88.9	1.38%	0.48%	0.55%	0.15%
Nitrospirales	*Thermodesulfovibrio aggregans*	86.8	1.66%	0.41%	0.38%	0.02%
